# Tubulointerstitial Nephritis and Uveitis Syndrome: A Case Report

**DOI:** 10.7759/cureus.66202

**Published:** 2024-08-05

**Authors:** Milliejoan Mongalo, Victoria Diaz, Andrew Kim, Jean Hou, Khalil Bourji

**Affiliations:** 1 Internal Medicine, Health Corporation of America (HCA) MountainView Hospital, Las Vegas, USA; 2 Pathology and Laboratory Medicine, Cedars-Sinai Medical Center, Los Angeles, USA; 3 Rheumatology, First Person Care Clinic, Las Vegas, USA

**Keywords:** anterior uveitis, nephritis, interstitial nephritis, uveitis, tinu

## Abstract

Tubulointerstitial nephritis and uveitis (TINU) syndrome is an uncommon autoimmune disorder that is defined by tubulointerstitial nephritis and uveitis. It is frequently underdiagnosed or goes unrecognized due to the challenges of accurately diagnosing the syndrome. TINU has mostly been seen among female pediatric patients with primarily bilateral anterior uveitis. However, screening for kidney disease often is overlooked; therefore, it is important for ophthalmologists, nephrologists, and rheumatologists to routinely screen for kidney disease and have TINU as a differential. We present a case of an adult female who had bilateral anterior uveitis for several years and then was found to have advanced chronic kidney disease, showing tubulointerstitial nephritis (TIN) features on renal biopsy.

## Introduction

Tubulointerstitial nephritis and uveitis (TINU) syndrome is an uncommon immune-mediated disease that was first identified by Dobrin et al. in 1975 [1]. TINU may be precipitated by medications (cidofovir, rifabutin, sulfonamides, bisphosphonates, and ibuprofen), infections (Epstein-Barr virus, herpes zoster virus, and toxoplasmosis), or maybe idiopathic [2,3]. TINU's prevalence has been variably reported, ranging from <0.1 to 2.0% in 'all age' cohorts and up to 2.2% in pediatric-specific cohorts [2,3]. TINU likely goes unrecognized and is underdiagnosed due to its heterogeneity within the uveitis spectrum. By definition, TINU is a diagnosis of exclusion, as there are various other etiologies of uveitis and tubulointerstitial nephritis (TIN). Herein, we present a case of a patient with bilateral anterior uveitis and TIN.

## Case presentation

A 29-year-old woman with a history of acute bilateral anterior uveitis diagnosed at age 14, resistant to steroid therapy, presented to us after being recently diagnosed with chronic kidney disease (CKD) stage 4, initially presumed to be secondary to chronic hypertension.

At onset, age 14, bilateral acute anterior uveitis presented with painful red eyes bilaterally. She was treated for many years with topical steroids and intermittently required high doses of oral prednisone to treat her several flares. At her most recent flare (three months before the renal injury), her ophthalmologic exam reported circumlimbal injection and anterior chamber cells (1-5 cells bilaterally). 

Initial labs showed complete urinalysis with elevated red blood cells (RBC) (10-20/HPF), white blood cells (WBC) (6-10/HPF), hyaline casts (0-5/HPF), squamous epithelial cells (6-10/HPF), and beta-2-microglobulin (36 mg/l). Her serum showed elevated creatinine (1.5 mg/dl), elevated erythrocyte sedimentation rate (ESR) (45 mm/h, normal <20), C-reactive protein (CRP) (9.4 mg/l, normal <8), and lysozyme (21 mcg/ml, normal 5-11). Given her elevated lysozyme level, a CT chest was done, which showed no findings suspicious for sarcoidosis. Despite her initial treatment with high-dose prednisone (30-40 mg/day) for about three months by her nephrologist, her renal function deteriorated and her creatinine rose to 3.09 mg/dl (estimated glomerular filtration rate (eGFR) 20), with remarkable proteinuria (urine protein creatine ratio of 1575 mg/g). Further lab studies included anti-nuclear antibodies (ANA), anti-neutrophil cytoplasmic antibodies (ANCA), HLA B27, rheumatoid factor (RF), cyclic citrullinated peptide antibodies (CCP), anti-smith antibody (SM), ribonucleoprotein antibody (RNP), Sjogren syndrome A and B antibodies (SS-A, SS-B), complement proteins (C3 and C4), and angiotensin-converting enzyme (ACE), all within normal limits. Previously, infectious etiology for her uveitis was ruled out with negative tuberculosis, syphilis, and cytomegalovirus (CMV) screening. In the context of her chronic anterior uveitis and newly diagnosed CKD, with the absence of potential connective tissues or autoimmune rheumatologic diseases (CTD/AIDs), a kidney biopsy was performed.

**Figure 1:**
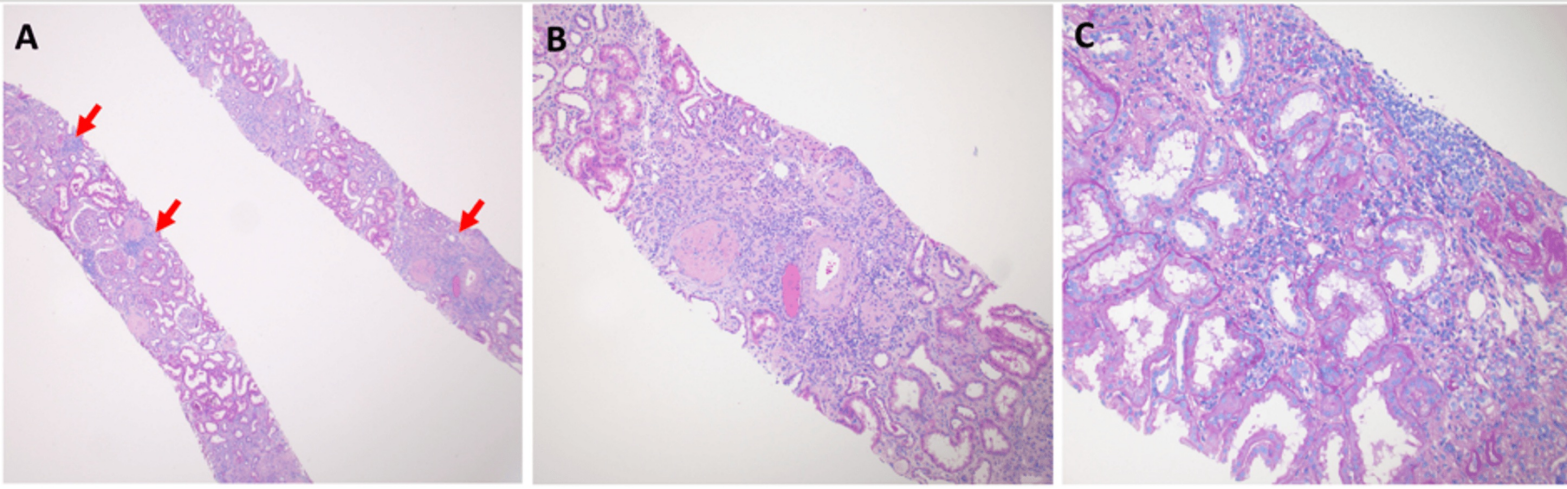
Renal biopsy pathology Light microscopy reveals areas of patchy interstitial inflammation (A: arrows, hematoxylin and eosin stain, magnification 100X). The inflammatory infiltrate is primarily localized within areas of tubulointerstitial scarring (B: hematoxylin and eosin, magnification 400X). The interstitial inflammatory infiltrate is mixed, with a combination of lymphocytes, plasma cells, and monocytes. Notably absent are neutrophils and eosinophils (C: Periodic acid Schiff stain, magnification 400X).

Renal biopsy revealed renal cortex with patchy areas of interstitial inflammation, primarily within areas of tubulointerstitial scarring. The inflammatory infiltrate was mixed with a combination of lymphocytes, plasma cells, and monocytes. There were no significant numbers of interstitial neutrophils (which helped rule out an infectious etiology) or eosinophils (which helped rule out a drug/medication-related allergic etiology). There was moderate arteriosclerosis with mild overall parenchymal scarring (Figure 1). The final diagnosis was chronic tubulointerstitial nephritis, the differential diagnosis for which typically includes infections, medications, reflux/obstruction, and autoimmune disease. However, given the clinical history of uveitis, the differential diagnosis was expanded to include rare etiologies such as TINU.

In the setting of stage 4 CKD, the patient was unable to use conventional disease-modifying anti-rheumatic drugs (cDMARDs) that are typically initially used to treat autoimmune uveitis, such as methotrexate or leflunomide. Therefore, adalimumab (a tumor necrosis factor-1 (TNF-1) inhibitor) was initiated. Within eight weeks of treatment, the patient was able to be weaned off the oral and topical steroids soon after, with clearance of her inflammatory ocular findings per her follow-up ophthalmologic exam.

## Discussion

Tubulointerstitial nephritis and uveitis (TINU) syndrome is a rare and underdiagnosed disorder, defined as the simultaneous occurrence of tubulointerstitial nephritis (TIN) and uveitis, in the absence of other systemic diseases that may cause uveitis and/or interstitial nephritis. After the initial description of two cases in 1975 by Dobrin et al., there have been more than 200 cases identified worldwide [1,2]. It has been reported with a mean age of 15 years, with females being more prominent at a 3:1 ratio [2,3,4]. Despite the recognition of TINU syndrome among the pediatric population, the clinical variability and ambiguity make it challenging to accurately diagnose and may delay treatment.

Although it's a rare entity, TINU's reported global prevalence varies among populations. In the all-age cohorts (without distinction between adult and pediatric cases), Okafor et al. analyzed about 17 retrospective or prospective studies reporting cases of TINU among patients visiting uveitis clinics, each including at least 500 patients, with prevalence ranging from 0.1 to 2%. On the other hand, four large cohorts of pediatric patients (younger than 18 years old), reported a higher prevalence (1.1-2.2%) [2,5-8].

After reviewing the clinical manifestations in 133 cases, Mandeville et al. proposed the first diagnostic criteria for TINU. It was found that interstitial nephritis precedes uveitis in 65% of cases, with 15% of cases occurring concurrent with uveitis, and uveitis preceding kidney disease in 20% [2,4] The most common manifestations that occur are fatigue, malaise, weight loss, fever, headache, abdominal or flank pain, arthralgias, and myalgias. Renal manifestations include impaired renal function in 90% of patients, with 64% of them having serum creatinine greater than 2.0 mg/dl. Other manifestations include sterile pyuria (55%), microhematuria (42%), renal polyuria, nocturia, and glucosuria (8%), as well as subnephrotic proteinuria and tubular defects. When comparing types of uveitis, the review demonstrated predominantly anterior uveitis in 80% of patients, with fewer cases of posterior or pan uveitis [2,4]. Ocular symptoms include ophthalmalgia, conjunctivitis, and vision impairment.

Furthermore, Standardization of Uveitis Nomenclature (SUN) was implemented in 2021, as the new guidelines for diagnosing TINU [2,9]. Both SUN and Mandeville classifications include similar clinical criteria, such as sudden bilateral anterior uveitis with concurrent TIN; TIN may be diagnosed via kidney biopsy pathology or by the combination of clinical presentation and laboratory investigations [2,9]. Urine beta-2 microglobulin and serum creatinine play a role in helping detect TIN and have been found to have high sensitivity and positive predictive value (PPV). Although both classifications are helpful in detecting TINU, increasing new cases have reported other clinical variabilities outside the “definition criteria,” which could lead to the need for expanding criteria further in the future, such as asymptomatic insidious uveitis and additional other posterior findings (chorioretinitis and neovascularization of the optic disc). Other challenges that arise when diagnosing TINU syndrome are ruling out other etiologies that may involve both ocular and TIN manifestations, such as sarcoidosis. According to SUN criteria, patients showing non-caseating granulomata histological features must be excluded; however, this would exclude all of Dobrin et al.’s cases and 13% of Mandeville et al.’s cases [2]. Therefore, many cases with TINU that have been reported may actually have underlying sarcoidosis or vice versa [2].

It is important to recognize the challenges of diagnosing TINU due to the limitations of classifications that focus solely on clinical phenotypes rather than distinct entities. Many patients diagnosed with uveitis are not screened for renal involvement, contributing to its lack of identification [2,3]. Therefore, TINU is an important differential to consider when encountering unexplained chronic or recurrent uveitis in order to diagnose and begin treatment early on.

## Conclusions

TINU syndrome is a rare disorder frequently overlooked and underdiagnosed. The clinical spectrum for diagnosing TINU is expanding, creating more challenges in detection. It is important to consider screening for kidney disease early on in patients with ocular manifestations. The patient in this case experienced a delay in the detection of renal disease. It is important to keep TINU syndrome as a differential diagnosis in patients who present with ocular manifestations and TIN in order to optimize treatment earlier.
